# BET bromodomain inhibitor JQ1 preferentially suppresses EBV-positive nasopharyngeal carcinoma cells partially through repressing c-Myc

**DOI:** 10.1038/s41419-018-0789-1

**Published:** 2018-07-09

**Authors:** Ning Li, Lu Yang, Xue-Kang Qi, Yu-Xin Lin, Xinhua Xie, Gui-Ping He, Qi-Sheng Feng, Ling-Rui Liu, Xiaoming Xie, Yi-Xin Zeng, Lin Feng

**Affiliations:** 1 Department of Experimental Research, Sun Yat-sen University Cancer Center, State Key Laboratory of Oncology in South China, Collaborative Innovation Center for Cancer Medicine, Guangzhou, 510060 China; 2 Department of Breast Oncology, Sun Yat-sen University Cancer Center, State Key Laboratory of Oncology in South China, Collaborative Innovation Center for Cancer Medicine, Guangzhou, 510060 China

## Abstract

The management of advanced nasopharyngeal carcinoma (NPC) remains a challenge. The ubiquitous nature of Epstein–Barr virus (EBV) infection in nonkeratinizing NPC has forced us to investigate novel drugs for NPC in the presence of EBV. In this study, we performed a small-scale screening of a library of compounds that target epigenetic regulators in paired EBV-positive and EBV-negative NPC cell lines. We found that bromodomain and extra-terminal (BET) inhibitor JQ1 preferentially inhibits the growth of EBV-positive NPC cells. JQ1 induces apoptosis, decreases cell proliferation and enhances the radiosensitivity in NPC cells, especially EBV-positive cells. Significantly, JQ1-induced cell death is c-Myc-dependent. Notably, RNA-seq analysis demonstrated that JQ1 represses TP63, TP53 and their targets. JQ1 also lessens the expression of PD-L1 in NPC. Moreover, the high potency of JQ1 in NPC cells was further confirmed in vivo in CNE2-EBV+ tumor-bearing mice. These findings indicate that JQ1 is a promising therapeutic candidate for advanced NPC.

## Introduction

Nasopharyngeal carcinoma (NPC) is a unique malignancy arising from the nasopharynx epithelium, and is highly endemic in south China and southeastern Asia^1^. Annually, approximately 86700 new cases and 50800 deaths are attributable to NPC worldwide^2^. With advances in radiotherapy and chemoradiotherapy, the 5-year survival of early or locoregionally advanced NPC is about 80%^3,4^. However, 15–30% of patients with NPC eventually develop distant metastasis, and the survival of these patients remains disappointing, with a median overall survival of only 20–30 months^4,5^. The non-keratinizing subtype of NPC constitutes most cases (>95%) in endemic regions, and shows the most consistent association with Epstein–Barr virus (EBV)^1,6^. After EBV infection, EBV latent genes can lead to genetic and epigenetic alterations, eventually resulting in the development of NPC^6^.

Epigenetics has been defined as potentially inheritable changes in gene expression that are not due to alterations in the primary sequence of DNA^7^. Epigenetic regulation plays a central role in control of cell fate and proliferation, and changes in epigenetic states have a major role in the development of multiple diseases, including cancer, metabolic disease, and inflammation^8^. The disease-associated epigenetic states are reversible, thus epigenetic-modulating agents, including small-molecule inhibitors of the epigenetic writers, readers and erasers, are being explored as candidate drugs^9^. Therapeutic exploitation of several epigenetic drugs, including DNA demethylating agents, HDAC inhibitors and bromodomain and extra-terminal (BET) inhibitors, has been made in multiple malignancies, and these drugs show great promise for clinical benefit^10,11^. Whether agents that target epigenetic regulators could have an antitumor effect on EBV-positive NPC cells remains to be explored.

A barrier to the development of targeted drugs for NPC lies in the shortage of authentic NPC cell lines that express EBV genome in long-term culture (There is currently only one cell line C666-1)^12,13^. Given the importance of EBV and epigenetics in NPC, we performed a small-scale screening of a library of compounds that target epigenetic regulators in paired EBV-positive and EBV-negative NPC cell lines. We indeed observed that JQ1 preferentially inhibits the growth of EBV-positive NPC cell lines both in vitro and in vivo. Our findings support clinical evaluation of JQ1 as a potential treatment option for advanced NPC.

## Results

### EBV-positive NPC cells are highly sensitive to JQ1

**Fig. 1:**
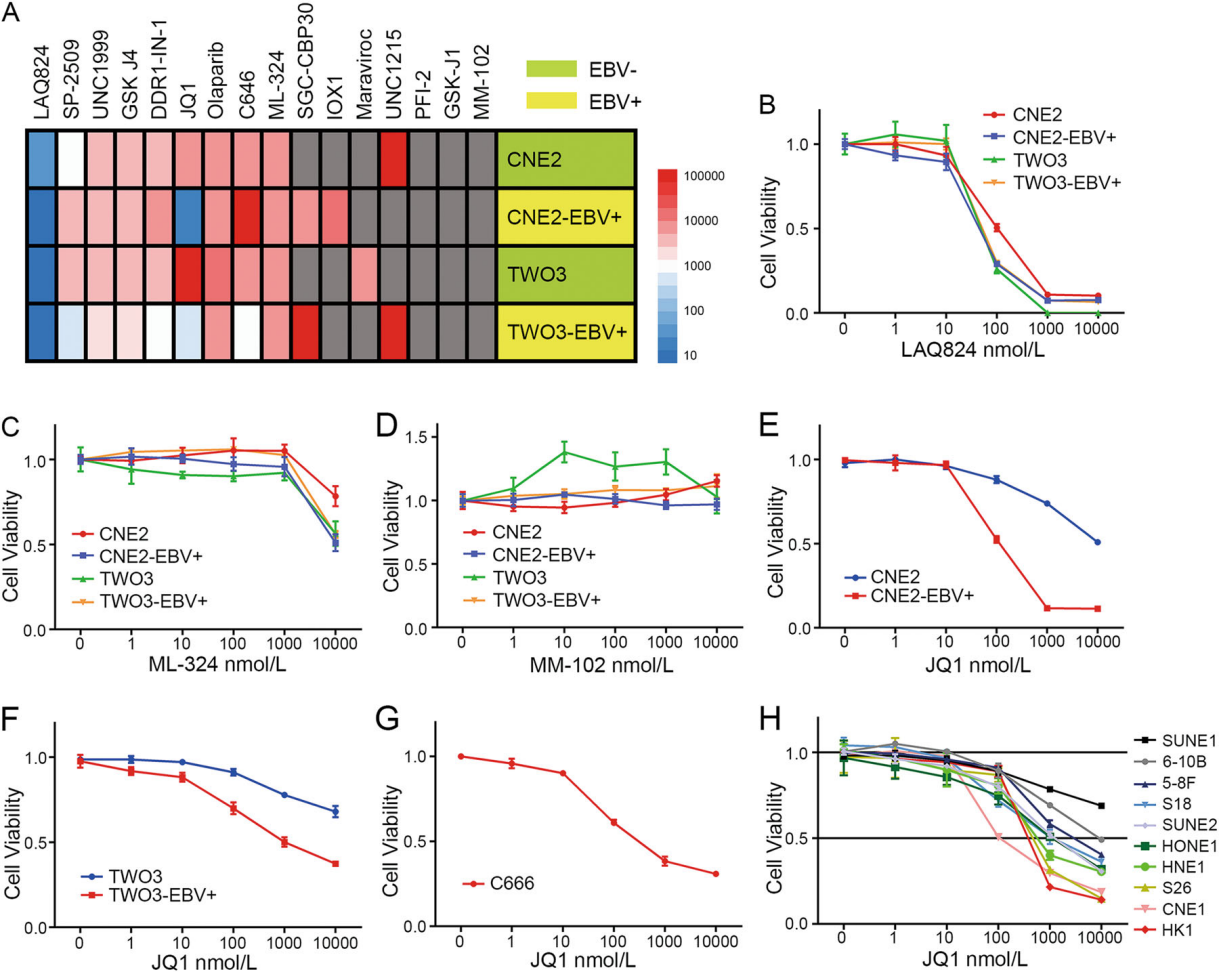
Identification of the selective compound for EBV+ NPC cells. **a** Heatmap of IC_50_ values of 16 inhibitors that target epigenetic regulators in CNE2-EBV−/+ and TWO3-EBV−/+ cell lines. Cells were treated with increasing concentrations of inhibitors for 72 h, and IC50 values were determined based on cell viability as measured by Cell-Titer GLO. “Gray” indicates unresponsiveness. **b** Cell viability of CNE2-EBV−/+ and TWO3-EBV−/+ cell lines upon treatment with increasing concentrations of LAQ824. **c** Cell viability of CNE2-EBV−/+ and TWO3-EBV−/+ cell lines upon treatment with increasing concentrations of ML-324. **d** Cell viability of CNE2-EBV−/+ and TWO3-EBV−/+ cell lines upon treatment with increasing concentrations of MM-102. **e** Cell viability of CNE2-EBV−/+ cell lines upon treatment with increasing concentrations of JQ1. **f** Cell viability of TWO3-EBV−/+ cell lines upon treatment with increasing concentrations of JQ1. **g** Cell viability of the C666 cell line upon treatment with increasing concentrations of JQ1. **h** Cell viability of the other 10 EBV-negative NPC cell lines upon treatment with increasing concentrations of JQ1. Data are presented as mean ± standard deviation (SD) of 3 independent experiments

To identify epigenetic-modulating agents that selectively inhibit the growth of EBV-positive NPC cells, we evaluated a panel of 16 small-molecule inhibitors that target epigenetic regulators in two pairs of EBV-positive and EBV-negative NPC cell lines, CNE2-EBV−/+ and TWO3−/+. The panel of small molecule inhibitors that target epigenetic regulators is illustrated in Table S1. Their targets included HDAC, LSD1, EZH2, BET, PARP, and H3K27 histone demethylase. From this small-scale screening, we found the BET inhibitor JQ1 showed a selective effect on EBV-positive NPC cell lines (Fig. 1a). LAQ824 and ML324 inhibited growth in both EBV-positive and EBV-negative NPC cell lines (Fig. 1b, c). All 4 cell lines were resistant to MM102 treatment (Fig. 1d). Only JQ1 inhibited the growth of CNE2-EBV+ and TWO3-EBV+ more potently than CNE2 and TWO3 (Fig. 1e, f). To determine the effect of JQ1 on a broader spectrum of NPC cell lines, we administered increasing concentrations of JQ1 to a panel of 11 NPC cell lines and two immortalized nasopharyngeal epithelial cell lines. The results showed that the EBV-positive cell line C666 was sensitive to JQ1 treatment (Fig. 1g). For the rest of the 10 EBV-negative NPC cell lines, their sensitivity to JQ1 varied (Fig. 1h). Interestingly, the most JQ1-sensitive EBV-negative NPC cell lines were two well-differentiated cell lines, CNE1 and HK1. NP69 and N5-tert were irresponsive to JQ1 treatment (Fig. S1).

### JQ1 induces apoptosis, decreases cell proliferation and enhances the radiosensitivity in NPC cells

**Fig. 2:**
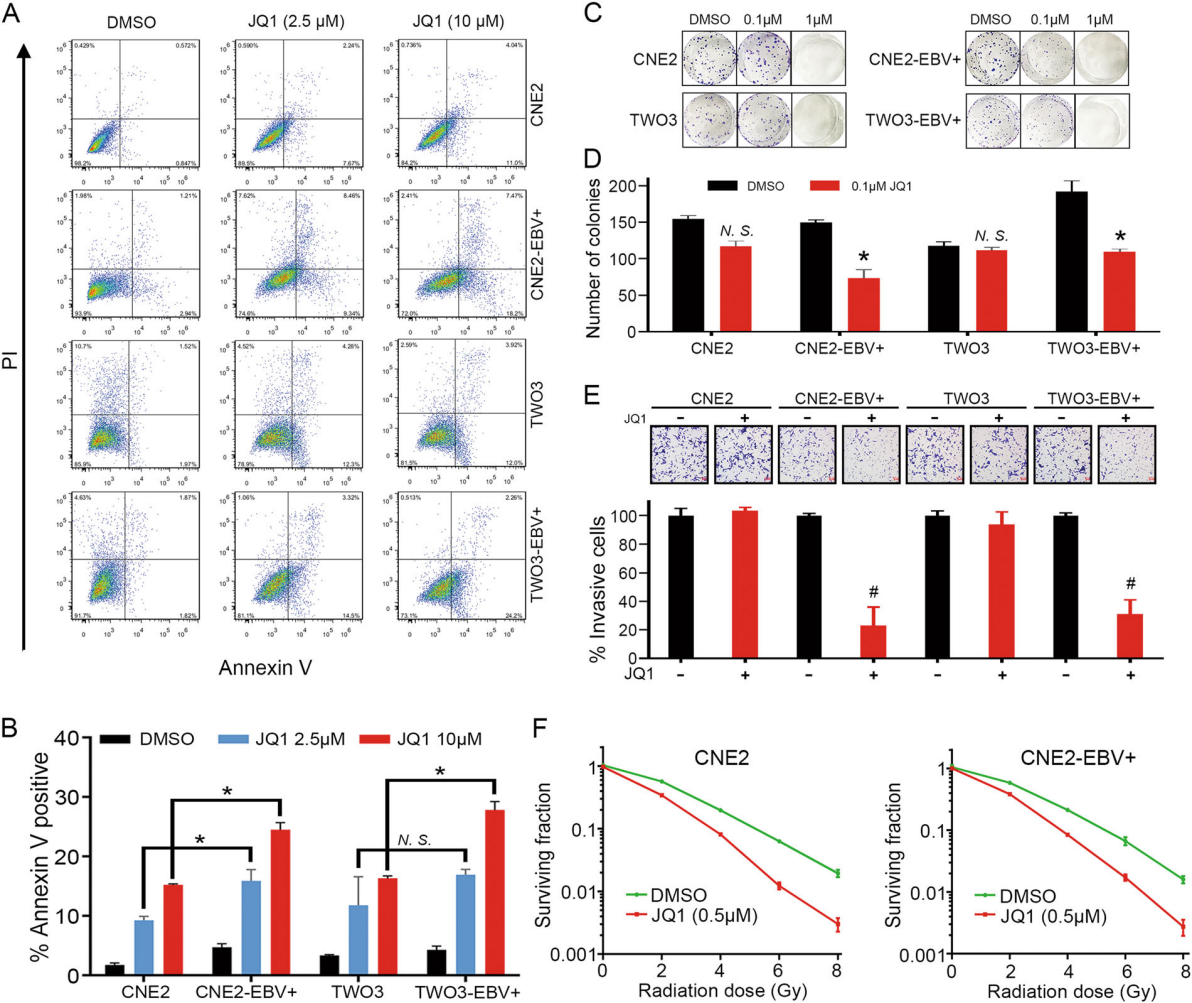
JQ1 induces apoptosis, blocks cell growth and enhances the radiosensitivity of NPC cells. **a** Annexin V and propidium iodide (PI) staining of CNE2-EBV−/+ and TWO3-EBV−/+ cells treated with DMSO, 2.5 μM JQ1 or 10 μM JQ1 for 48 h. Experiments were repeated 3 times and representative images are shown. **b** Quantification of Annexin V positive cells shown in **a**. Data are presented as mean ± SD of 3 independent experiments. **c** Colony formation assays of CNE2-EBV−/+ and TWO3-EBV−/+ cell lines. Cells were cultured in the presence of DMSO, 0.1 μM JQ1 or 1 μM JQ1 for 10–14 days followed by crystal violet staining. Representative photographs are shown. **d** Number of colonies for CNE2-EBV−/+ and TWO3-EBV−/+ cells treated with DMSO or 0.1 μM JQ1. **e** Invasion assays of CNE2-EBV−/+ and TWO3-EBV−/+ cell lines with or without JQ1 treatment. JQ1 was used at 50 nM. Quantification of invasive cells shown below. **f** Clonogenic cell surviving curves of CNE2 and CNE2-EBV+ cells that were pretreated with JQ1 (500 nM) or DMSO for 24 h and then exposed to 2, 4, 6 or 8 Gy of X-ray irradiation. 48 h after irradiation, media were replaced by fresh drug-free media. Colony-forming efficiency was determined 10–14 days later. The data are reported as mean ± SD of 3 independent repeats. *P < 0.05; ^#^*P* < 0.01

JQ1-induced cell death was then confirmed by Annexin V/PI staining. The results showed that JQ1 treatment in the concentration of 2.5 μM or 10 μM induced a significant increase in the number of Annexin-positive cells, suggesting that these cells were undergoing apoptosis (Fig. 2a). Of note, JQ1 treatment induced more Annexin-positive cells in EBV-positive NPC cells than in EBV-negative cells (Fig. 2b). The administration of JQ1 in the concentration of 1 μM inhibited the clonogenic growth of all cell lines, whereas 0.1 μM JQ1 only inhibited the clonogenic growth of CNE2-EBV+ and TWO3-EBV+ cell lines (Fig. 2c, d). Furthermore, JQ1 treatment also led to significant inhibition of the invasion of CNE2-EBV+ and TWO3-EBV+ cells (Fig. 2e). The above results were in consistency with their response pattern observed in cell viability assays.

To determine the effect of JQ1 on the radiosensitivity of NPC, CNE2 and CNE2-EBV+ cells were pretreated with JQ1 (0.5 μM) or DMSO for 2 days and were then exposed to increasing doses of ionizing radiation (IR). As shown in Fig. 2f, treatment with JQ1 led to significant radiosensitization in CNE2 and CNE2-EBV+ cell lines. The dose enhancement ratios (DERs) at a surviving fraction of 0.5 were 1.415 for CNE2 and 1.358 for CNE2-EBV+ cells.

**Fig. 3:**
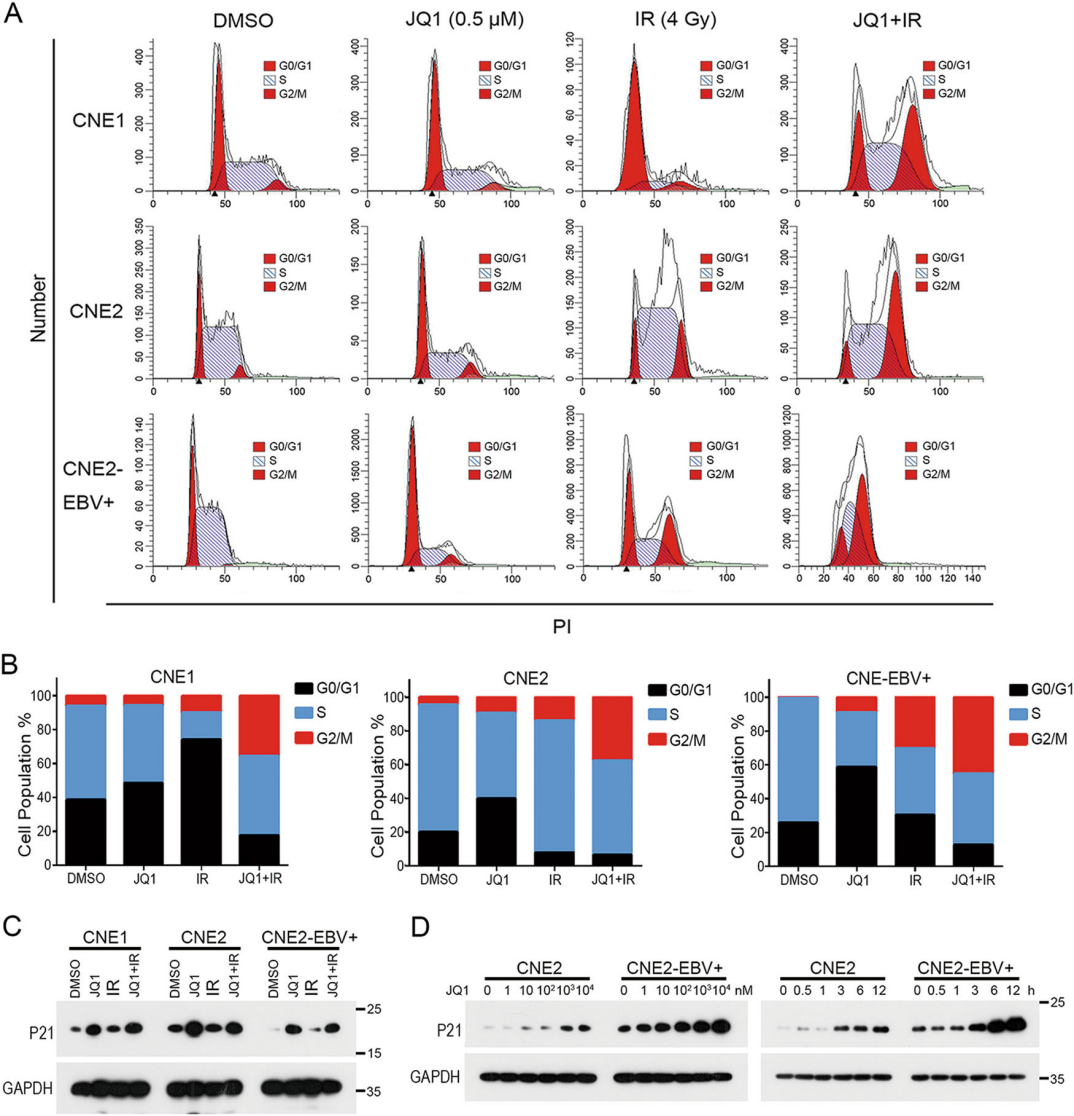
JQ1 induces G2/M cell cycle arrest of NPC cells after irradiation. **a** Cell cycle analysis of CNE1, CNE2, and CNE2-EBV+ cells after treatment with DMSO, JQ1 (500 nM), IR (4 Gy), or 500 nM JQ1 for 48 h followed by IR of 4 Gy. **b** Quantification of each cell cycle phase in CNE1, CNE2, and CNE2-EBV+ cells in each group shown in **a**. **c** Western blot analysis of antibodies against p21 of CNE1, CNE2, and CNE2-EBV+ cells after treatment with DMSO, JQ1 (500 nM), IR (4 Gy), or 500 nM JQ1 for 48 h followed by IR of 4 Gy. GAPDH served as a loading control. **d** Western blot analyses of the concentration- and time-dependent effect of JQ1 treatment on p21 expression in CNE2-EBV−/+ cell lines

To evaluate the effects of JQ1 on cell cycle arrest of NPC cells with or without IR, CNE1, CNE2 and CNE2-EBV+ cells were treated with either DMSO, 0.5 μM JQ1, IR (4 Gy), or 0.5 μM JQ1 for 48 h followed by irradiation with 4 Gy. Compared with either DMSO, JQ1 or IR alone, the combination of JQ1 and IR led to increased rates of G2/M arrest and a dramatic decrease in G0/G1 and S phases in both cell lines (Fig. 3a, b). Considering that p21 plays a critical role in cell cycle and DNA repair, we examined the protein level of p21 with or without JQ1 treatment. JQ1 alone or with IR led to the increase of p21 in NPC cells, suggesting the elevation of p21 is associated with JQ1-mediated cell cycle arrest, but not IR-mediated cell cycle arrest (Fig. 3c). The increase of p21 upon JQ1 treatment was observed to be concentration- and time-dependent (Fig. 3d). We further assessed whether JQ1 could promote apoptosis of NPC cells after IR. CNE2 and CNE2-EBV+ cells were treated with DMSO, 0.5 μM JQ1, IR (4 Gy), or 0.5 μM JQ1 for 48 h followed by exposure to 4 Gy IR. The results showed that the combination treatment induced a significant increase of apoptosis in both cell lines. The rate of Annexin-positive cells was as high as 34.5% for CNE2-EBV+ with the combination treatment of JQ1 (0.5 μM) and IR (4 Gy), whereas it was 13.9% for CNE2 (Fig. S2). These results suggest that JQ1 promotes apoptosis and G2/M cell cycle arrest of NPC cells after IR, especially for EBV+ NPC cells.

### JQ1-induced cell death is c-Myc-dependent in NPC

**Fig. 4:**
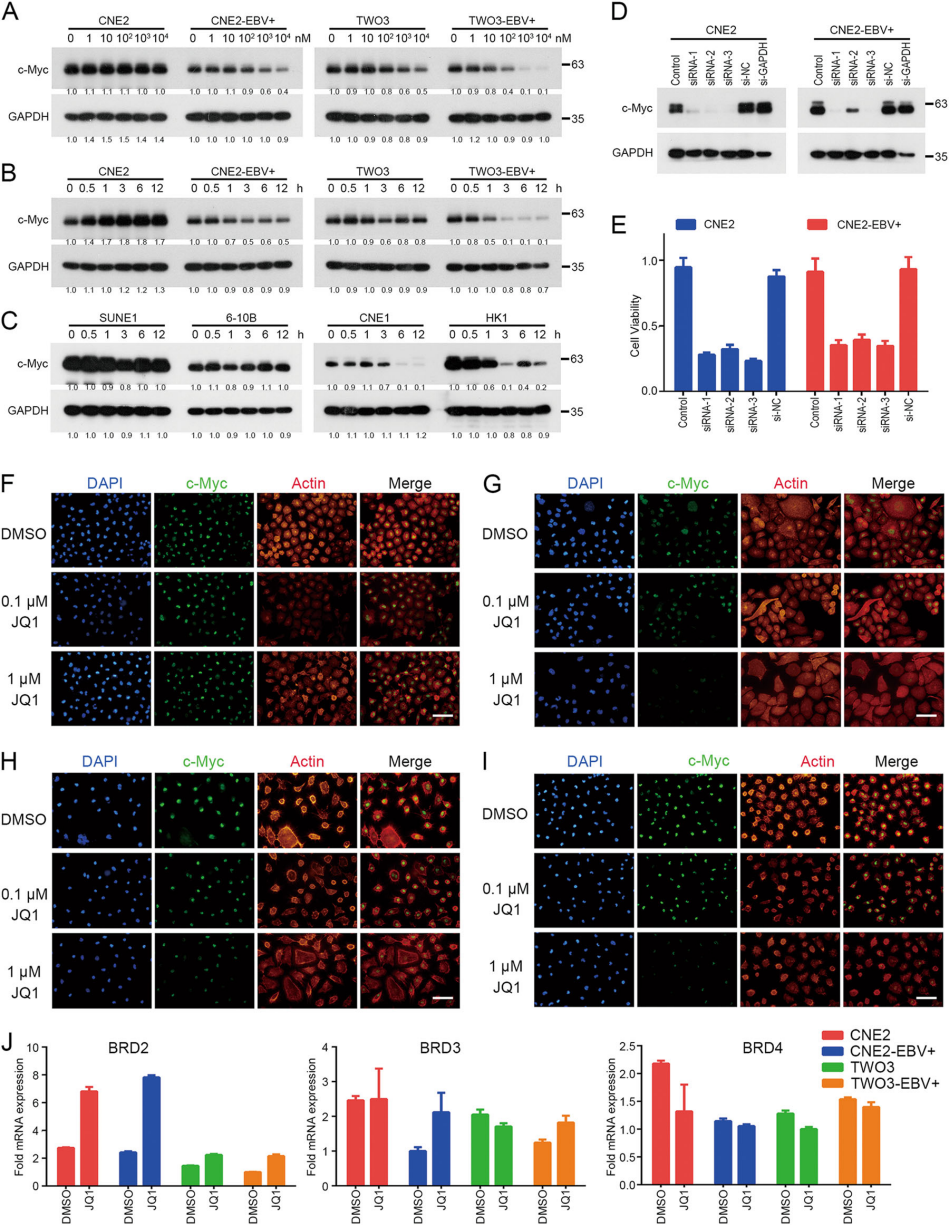
JQ1-induced cell death is c-Myc-dependent in NPC. **a** CNE2-EBV−/+ and TWO3-EBV−/+ cells were treated with indicated concentrations of JQ1 for 24 h and analyzed for c-Myc level by western blot analysis. GAPDH served as a loading control. **b** CNE2-EBV−/+ and TWO3-EBV−/+ cells were treated with 1 μM JQ1 for 0, 0.5, 1, 3, 6, and 12 h and were analyzed for c-Myc level by western blot analysis. GAPDH served as a loading control. **c** Western blot analyses of the time-dependent effect of JQ1 treatment on c-Myc expression in SUNE1, 6-10B, CNE1 and HK1 cells. JQ1 was used at 1 μM. **d** Western blot analysis of RNAi efficiency of c-Myc siRNA in control CNE2-EBV−/+ cells and those transfected with c-Myc-targeting siRNA or negative control (NC) siRNA. Cell lysates were collected 48 h after transfection. **e** Cell viability of CNE2-EBV−/+ cells and those transfected with c-Myc-targeting siRNA or NC siRNA. Cell viability was measured 72 h after transfection. **f**–**i** Immunofluorescence images of **f** CNE2, (**g**) CNE2-EBV+, (**h**) TWO3, and (**i**) TWO3-EBV+ cells stained with antibodies against c-Myc. Actin was stained by FITC phalloidin and nuclei were stained by DAPI. Scale bar = 50 μm. **j** qRT–PCR analyses of BRD2, BRD3, and BRD4 in CNE2-EBV−/+ and TWO3-EBV−/+ cell lines treated with 1 μM JQ1 for 24 h. Results were normalized to GAPDH. The data are presented as mean ± SD of 3 independent repeats

The efficacy of JQ1 has been attributed mainly to its ability to suppress the expression of c-Myc in many malignancies^14^. However, because cancers from different cells of origin vary drastically in their epigenetic landscapes, whether the efficacy of JQ1 on NPC cells relies on the suppression of c-Myc remains to be explored. To this end, western blot analysis of antibodies against c-Myc and GAPDH was performed. The results showed that JQ1 led to the decrease of c-Myc in CNE2-EBV+ and TWO3-EBV+ cell lines in a concentration- and time-dependent manner (Fig. 4a, b). However, c-Myc protein levels remained unchanged, or changed only slightly, after JQ1 treatment in CNE2 and TWO3 cell lines (Fig. 4a, b). Surprisingly, JQ1 led to the decrease of c-Myc in sensitive EBV-negative cell lines CNE1 and HK1, but not in insensitive EBV-negative cell lines SUNE1 and 6-10B (Fig. 4c).

Next, we performed knockdown experiment of c-Myc to determine if suppression of c-Myc phenocopies the effects of JQ1 treatment. The effect of c-Myc knockdown was evaluated by 3 siRNAs (Fig. 4d). The knockdown of c-Myc significantly reduced the viability of CNE2 and CNE2-EBV+ cells (Fig. 4e). We then confirmed the western blot analysis results of protein level changes of c-Myc in CNE2-EBV−/+ by immunofluorescence (Fig. 4f-i). The results showed that treatment with 1 μM JQ1 led to significant reduction of c-Myc in CNE2-EBV+ and TWO3-EBV+ cells, but not in CNE2 and TWO3 cells (Fig. 4f-i). In addition, after JQ1 treatment, the mRNA levels of BRD2 increased, but BRD3 and BRD4 changed inconsistently or remained unchanged (Fig. 4j). Taken together, these findings indicate that c-Myc down-regulation is a requirement for the antineoplastic effects of JQ1 in NPC.

### JQ1 represses TP63, TP53 and their targets, and PD-L1 in NPC

**Fig. 5:**
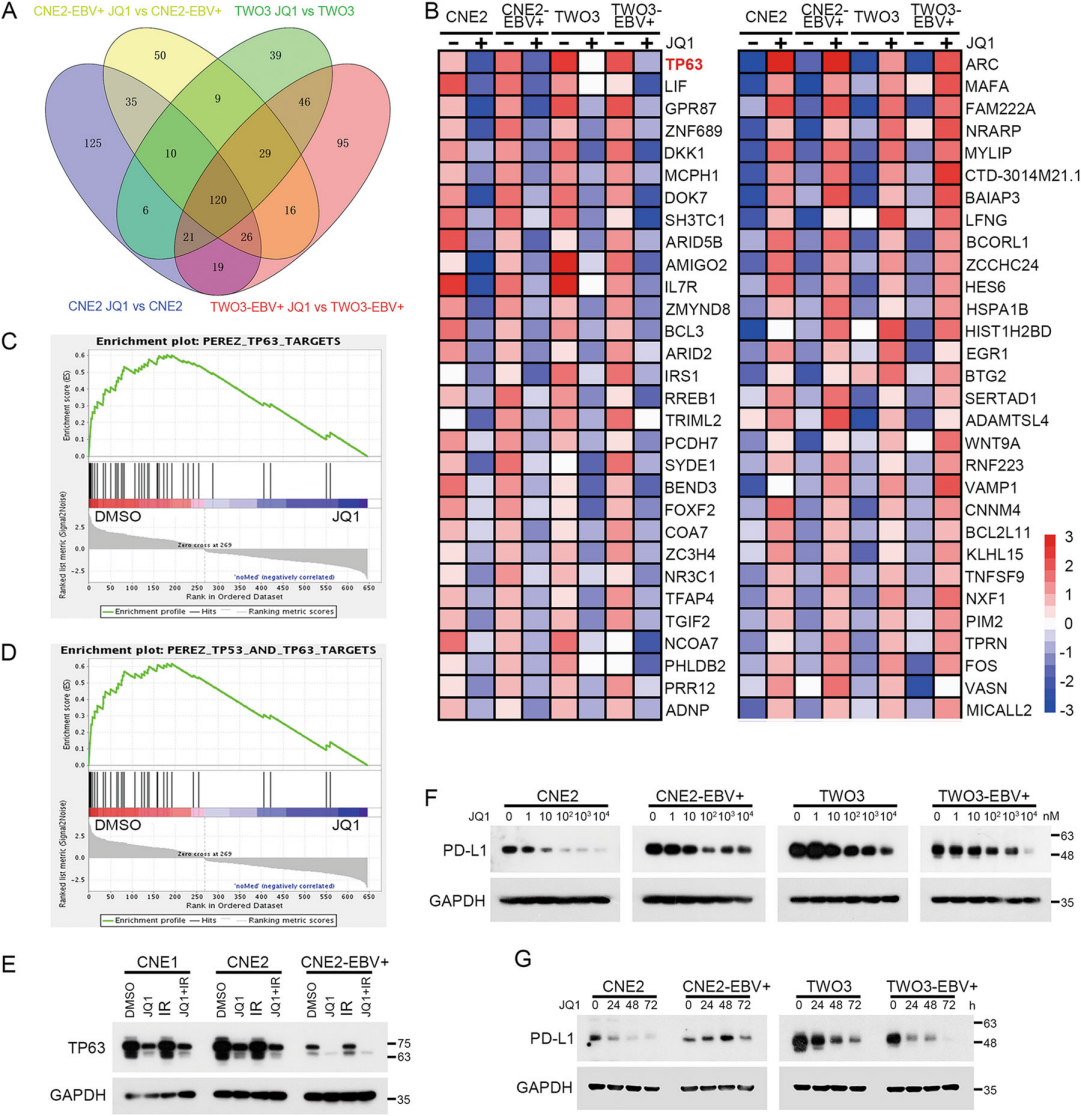
JQ1 represses TP63, TP53 and their targets, and PD-L1 in NPC. **a** Venn diagram showing the overlap of significantly altered genes after exposure to 1 μM JQ1 for 3 h in CNE2-EBV−/+ and TWO3-EBV−/+ cell lines. **b** Heatmap representation of the top-30 down- and up-regulated genes following 3 h of 1 μM JQ1 treatment of all CNE2-EBV−/+ and TWO3-EBV−/+ cell lines. Data are presented as row normalized. **c** GSEA showing downregulation of TP63-dependent gene sets in the transcriptional profiles of NPC cell lines treated with JQ1. **d** GSEA displaying downregulation of TP53- and TP63-dependent gene sets in the transcriptional profiles of NPC cell lines treated with JQ1. **e** Western blot analysis of antibodies against TP63 of CNE1, CNE2, and CNE2-EBV+ cells after treatment with DMSO, JQ1 (500 nM), IR (4 Gy), or 500 nM JQ1 for 48 h followed by IR of 4 Gy. GAPDH served as a loading control. **f** CNE2-EBV−/+ and TWO3-EBV−/+ cells were treated with indicated concentrations of JQ1 for 24 h and were analyzed for PD-L1 level by western blot analysis. GAPDH served as a loading control. **g** CNE2-EBV−/+ and TWO3-EBV−/+ cells were treated with 1 μM JQ1 for 0, 24, 48 and 72 h and analyzed for PD-L1 level by western blot analysis. GAPDH served as a loading control

Since JQ1 can enhance the radiosensitivity of both EBV- and EBV+ NPC cells, RNA-seq analysis of CNE2-EBV−/+ and TWO3-EBV−/+ cells treated with or without JQ1 was performed to seek the relevant targets. The results showed that there were 120 genes whose expression was changed 2-fold or more in all four cell lines (Fig. 5a). Ranking these genes by their differential expression scores revealed that TP63 was the top gene that was strongly down-regulated by JQ1 treatment (Fig. 5b). Furthermore, gene set enrichment analysis (GSEA) of genes down-regulated by JQ1 revealed significant enrichment for genes having DNA binding motifs for TP63 and TP53 (Fig. 5c, d). Down-regulation of TP63 was further confirmed by western blot analysis (Fig. 5e). The protein level of TP63 was decreased upon JQ1 alone or with IR treatment in CNE1, CNE2, and CNE2-EBV+ cells (Fig. 5e).

To further investigate the precise mechanism of the preferential sensitivity of EBV+ cell lines to JQ1, RNA-seq analysis of the differences between EBV+ cell lines and EBV- cell lines with or without JQ1 treatment was performed (Fig. S3). Surprisingly, from this analysis c-Myc was not among the top 30 genes uniquely down-regulated by JQ1 in EBV+ cell lines. Thus, although c-Myc down-regulation probably plays a role in NPC’s response to JQ1, additional factors may operate on the resistance to JQ1. Previous reports have demonstrated that JQ1 suppresses the expression of PD-L1 and limits tumor progression in ovarian cancer^15^. To test whether JQ1 can down-regulate the expression of PD-L1 in NPC, CNE2-EBV−/+ and TWO3-EBV−/+ cells were treated with JQ1. Western blot analysis showed that the protein level of PD-L1 was decreased in the four cell lines in a concentration-dependent manner (Fig. 5f), and in CNE2, TWO3 and TWO3-EBV+ in a time-dependent manner (Fig. 5g).

### JQ1 inhibits NPC xenograft growth in vivo

**Fig. 6:**
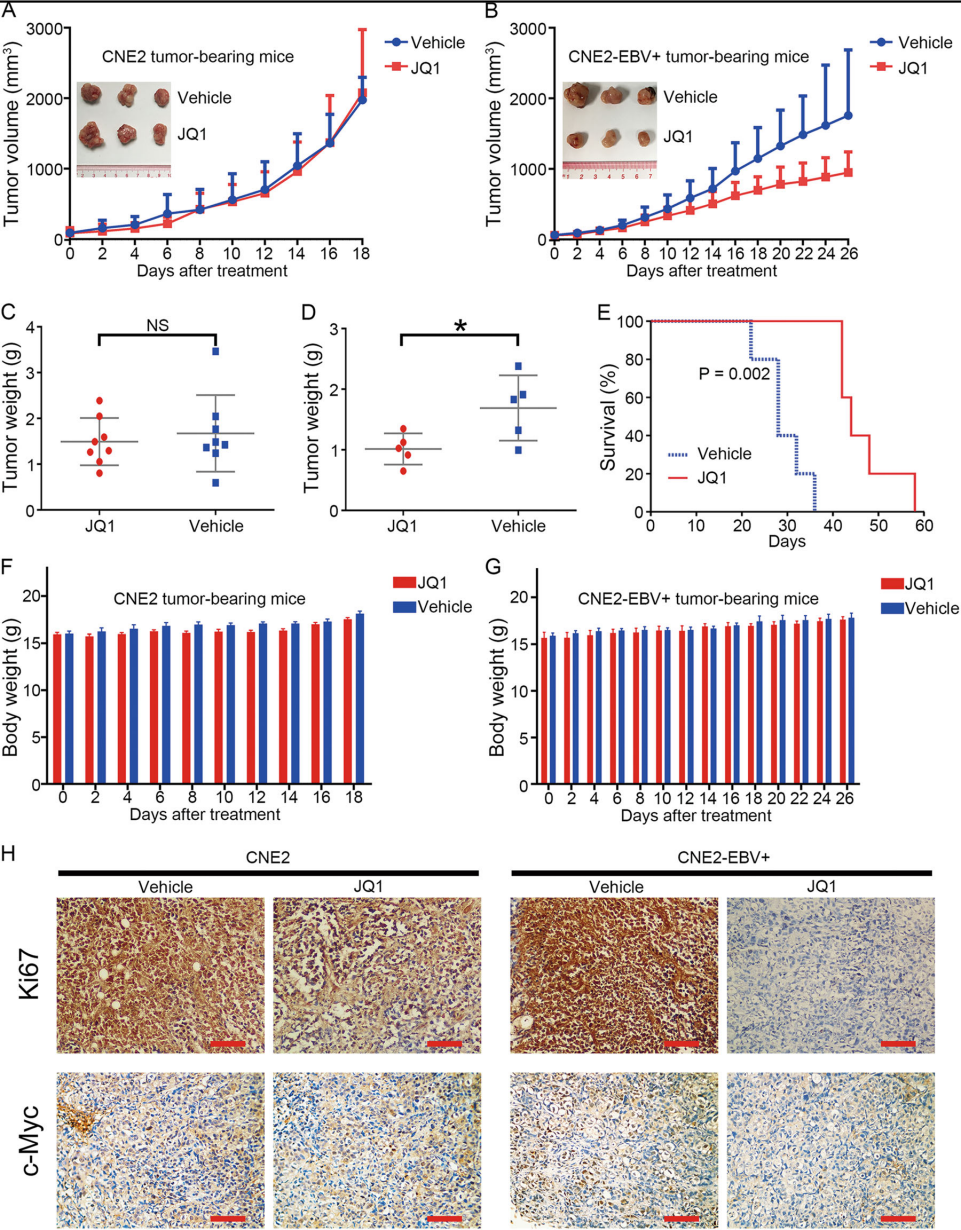
JQ1 exhibits antitumor activity in nasopharyngeal carcinoma xenograft models. **a**, **b** Changes in tumor volume in JQ1 group and vehicle group of **a** CNE2 tumor-bearing mice and **b** CNE2-EBV+ tumor-bearing mice. Data are shown as mean tumor volume ± SD. **c**, **d** Tumor weight differences between JQ1 group and vehicle group of **c** CNE2 tumor-bearing mice and **d** CNE2-EBV+ tumor-bearing mice. *P < 0.05. **e** Kaplan–Meier survival curve of immunodeficient mice injected subcutaneously with CNE2-EBV+ cells and treated with JQ1 (50 mg/kg) or vehicle control (six mice/group). Mice were sacrificed when tumor volume reached 2000 mm^3^. Significance was performed by Log-Rank (Mantel–Cox) test. **f, g** Changes in body weight in JQ1 group and vehicle group of **f** CNE2 tumor-bearing mice and **g** CNE2-EBV+ tumor-bearing mice. **h** Tumor sections from JQ1-treated and vehicle-treated CNE2 tumor-bearing mice and CNE2-EBV+ tumor-bearing mice were immunostained with Ki67 or c-Myc antibody. All images were collected at the same magnification. Scale bar = 100 μm

To probe the in vivo efficacy of JQ1 on NPC growth, we tested its effects in a subcutaneous tumor model. In CNE2 tumor-bearing mice, treatment with intraperitoneal injection of JQ1 did not inhibit NPC tumor growth (Fig. 6a). However, in CNE2-EBV+ tumor-bearing mice, measurement of tumor volume over time course revealed much slower growth in the JQ1 group compared with the vehicle-treated group (Fig. 6b). JQ1 treatment did not reduce tumor weight in CNE2 tumor-bearing mice, whereas resulted in reduced tumor weight in CNE2-EBV+ tumor-bearing mice (Fig. 6c, d). JQ1 treatment also led to significant survival benefit over the control in CNE2-EBV+ tumor-bearing mice (Fig. 6e). JQ1 was well tolerated, since no significant weight loss in mice was observed (Fig. 6f, g). Histologic analysis of harvested tumors revealed that JQ1 led to the decrease of Ki-67 and c-Myc expression in CNE2-EBV+ xenografts (Fig. 6h). In contrast, JQ1 marginally affected Ki-67 expression and failed to affect c-Myc expression in CNE2 xenografts (Fig. 6h). These data show potent in vivo anti-tumor activity of single-agent JQ1 against EBV+ NPC cells.

## Discussion

Chemotherapy continues to feature as the standard treatment for metastatic NPC, albeit the administration of chemotherapy has run into the bottleneck in NPC^4^. Recently, many efforts have been made to exploit strategies to develop novel therapies for NPC, and this has led to the development of epidermal growth factor receptor (EGFR) inhibitors, vascular endothelial growth factor (VEGF) inhibitors and immunotherapeutic drugs^1,16^. However, a barrier to the development of novel drugs for NPC lies in the shortage of authentic NPC cell lines that express EBV genome in long-term culture. EBV has been discovered for more than 50 years, and it has been linked to the development of NPC^6^. The ubiquitous nature of EBV infection in nonkeratinizing NPC has forced us to investigate novel drugs for NPC in the presence of EBV.

Here, we identified JQ1 as a promising drug candidate for the treatment of NPC. Using a small-scale small-molecule screening approach, we observed that EBV+ NPC cells were preferentially sensitive to JQ1 treatment. We also observed that JQ1-induced cell death is c-Myc-dependent, and JQ1 represses TP63, TP53 and their targets, and PD-L1 in NPC. The high potency of JQ1 in NPC cells was further confirmed in vivo in CNE2-EBV+ tumor-bearing mice.

BET family members (BRD2, BRD3, BRD4, and BRDT) are “readers” of acetyl lysine residues and have a crucial role in transcriptional elongation^14,17^. BET proteins have been identified in the regulation of oncogenic transcriptional programs that lead to oncogenic fusion proteins, and lead to cancer ultimately^14^. JQ1 is a potent competitive inhibitor of acetylation of lysine residues for BET, and it was initially proven to be effective in nuclear protein in testis (NUT) midline carcinoma (NMC), a cancer driven by the BRD4-NUT fusion protein^18^. Subsequently, JQ1 and other BET inhibitors have been shown to be effective in a variety of hematologic malignancies^19–24^ and solid tumors^25–32^. Our results demonstrated that JQ1 is effective in EBV+ NPC cells. In our study, BRD2 was upregulated by JQ1. The upregulation of BRD2 was previously shown in a novel BET inhibitor, OTX015^33^. JQ1 may down-regulate transcriptional repressors or other negative regulators of BRD2.

The efficacy of BET inhibitors has been attributed mainly to their ability to preferentially repress transcription of c-Myc^14^. c-Myc is a multifunctional regulator gene of cell proliferation and apoptosis, and abnormal activation of c-Myc results in the formation of cancer^34^. In NPC, c-Myc is amplified and upregulated after depletion of ARID1A, which is the most frequently altered gene in NPC^35^. Although the efficacy of BET inhibitors has been attributed to c-Myc downregulation in many malignancies, including acute myeloid leukemia^19^, multiple myeloma^20^, Burkitt’s lymphoma^21^, MLL-fusion leukemia^22^, acute lymphoblastic leukemia^23^, diffuse large B cell lymphoma^24^, glioblastoma^27^, melanoma^28^, prostate cancer^29^, colon cancer^30^, and thyroid cancer^32^, but not in lung adenocarcinoma^25^ and breast cancer^31^. We observed that c-Myc down-regulation is a requirement for the antitumor effects of JQ1 in NPC and knockdown of c-Myc can phenocopy JQ1 treatment. In lymphoblastoid cell lines, all oncogenic EBV nuclear antigens (EBNAs) were at the enhancer site of MYC oncogene, which was designated as an EBV super-enhancer-associated gene^36^. This is probably the main reason for the preferential sensitivity of EBV+ NPC cells to JQ1 treatment. Of note, EBV- cell lines CNE1 and HK1 were sensitive to JQ1 treatment and JQ1 led to the decrease of c-Myc in CNE1 and HK1. CNE1 and HK1 are both well-differentiated cell lines^12^. Thus, the efficacy of JQ1 might be associated with the differentiation state of NPC. Although the precise mechanism remains to be determined, our results suggest that JQ1-induced cell death is c-Myc-dependent in NPC cells.

To test the effect of JQ1 on gene transcription in both EBV+ and EBV- NPC cells, we performed RNA-seq analysis. TP63 mRNA and protein levels were down-regulated by JQ1, and GSEA analysis identified significant enrichment of TP63, TP53 and their targets within the set of JQ1-repressed genes in both cell lines. TP63 is a member of the TP53 family and has important functions in embryonic development and skin homeostasis^37^. However, the role of TP63 in tumorigenesis remains elusive. Although evidence suggests that TP63 functions as a tumor suppressor, TP63 is overexpressed in many cancers, including head and neck cancers^37^. p21 represents a major target of TP53 and plays a major role in DNA damage and cell cycle arrest^38^. We observed that JQ1 led to the increase of p21 while it led to the decrease of TP63, suggesting JQ1 induced radiosensitization effects in NPC are associated with p21 up-regulation. The recent successes of immunotherapy have dramatically transformed cancer care^39^. As reported previously^15^, our results showed that JQ1 suppresses the expression of PD-L1. The possible reason may be largely related to the close relationship between c-Myc and PD-L1. c-Myc regulates the expression of PD-L1 and therapies suppressing c-Myc expression may restore antitumor immunity^40^. Our observations raise the possibility of PD-L1 blockade with a small-molecule approach in NPC.

Of note, mice CNE2-EBV+ tumors were sensitive to JQ1 treatment, whereas CNE2 tumors were resistant to JQ1. The in vivo results were in consistency with their in vitro response pattern. As previously reported^21,29^, the administration of JQ1 did not result in weight loss or severe adverse effects in mice in our study, suggesting JQ1 is well tolerated. In conclusion, we report the preferential potency of JQ1 on EBV-positive NPC cells in vitro and in vivo, establishing a rationale for clinical investigation of JQ1 as a drug candidate for the treatment of advanced NPC.

## Materials and methods

### Cell lines and reagents

Cell lines used in this study (NP69, N5-tert, CNE1, CNE2, S18, S26, HONE1, HNE1, HK1, SUNE1, 5–8F, 6-10B, SUNE2, and C666) were maintained and conserved in our laboratory. CNE2-EBV+ and N5-tert were gifts from Prof. Mu-Sheng Zeng. TWO3 and TWO3-EBV+ were kindly provided by Prof. Jiang Li. The immortalized nasopharyngeal epithelial cell lines NP69 and N5-tert were cultured in a keratinocyte serum-free medium (Gibco, CA) supplemented with bovine pituitary extract. The human NPC cell lines were cultured in RPMI-1640 medium (Invitrogen, CA) supplemented with 10% fetal bovine serum (Gibco) at 5% CO_2_, 37 °C. The panel of small molecule inhibitors that target epigenetic regulators (Table S1) was purchased from APExBIO company (TX) and Selleck company (China). The following primary antibodies were used: c-Myc (CST, #13987), TP63 (Abcam, #ab124762), p21 (CST, # 2947), PD-L1 (CST, #13684), Ki67 (Abcam, #ab15580), and GAPDH (Proteintech, 60004-1-Ig).

### Cell viability assay

Cell viability was assessed by Cell-Titer GLO (Promega, G7572; WI) based on the manufacturer’s specifications. Briefly, cells were seeded in opaque 96-well plates (2000–10000 cells/well) in 100 μL media per well, and were allowed to adhere overnight. The next day, cells were treated with increasing doses of small molecule inhibitors or DMSO for up to 72 h. After addition of 100 μL Cell-Titer GLO reagent, cells were incubated at room temperature for 10 min. Absorbance was read with a GloMax luminometer (Promega). Cell viability was expressed graphically as mean ± SD of absorbance and IC50 values were calculated by nonlinear regression analysis with GraphPad Prism 6.

### Annexin V staining

Apoptosis was determined by double staining of Annexin V–propidium iodide (PI) with an Annexin V–PI apoptosis detection kit (Keygen Biotech, KGA108; Nanjing, China) according to the manufacturer’s instructions. Briefly, cells were treated as indicated. Cells were then harvested by 0.25% trypsin without EDTA, washed twice with PBS and resuspended in 500 μL binding buffer solution. After addition of 5 μL Annexin V and 5 μL PI, cells were incubated at room temperature for 15 min in the dark. Then, stained cells were subjected to analyses by a Gallios flow cytometry (Beckman–Coulter) within 1 h.

### Colony formation assay

Cells were seeded in 6-well dishes in triplicate at 500–2000 cells per well. After attachment overnight, the cells were treated as indicated. After 10–14 days of incubation, they were washed with cold PBS, fixed in methanol, and stained with crystal violet. They were then air-dried before being photographed. For radiosensitization studies, the cells were treated with DMSO or JQ1 and irradiated with indicated doses of radiation 24 h after drug or vehicle exposure. At 48 h after irradiation, JQ1 was washed off. The colonies were counted, and surviving fractions were calculated as follows: average number of colonies/(number of cells plated × plating efficiency). Plating efficiency was calculated as the average number of colonies/the number of cells plated. We then calculated the dose enhancement ratio (DER) as the ratio of the radiation dose (Gy) without JQ1 to the radiation dose (Gy) with JQ1 at the same surviving fraction.

### Cell cycle analysis

Cells were seeded in 6-well plates and treated as indicated for 48 h. The cells were then washed with PBS and fixed in 70% cold ethanol overnight at 4 ℃. They were then washed again with PBS and stained with 120 μL of staining solution containing PI (20 μg/mL; Sigma), RNase A (25 μg/mL; Sigma), and 0.1% Triton X-100 in PBS. The cells were incubated at room temperature for 30 min and analyzed by a Gallios flow cytometry (Beckman–Coulter).

### Invasion assay

NPC cells were treated with 50 nM JQ1 or DMSO for 24 h, and then they were seeded in transwell chambers pre-coated with Matrigel (BD Biosciences). For JQ1 group, 50 nM JQ1 was added to both upper and lower chambers. After incubation for 48 h, the transwell chambers were gently cleaned with cotton swabs and stained with crystal violet, and then photographed.

### Western blots

NPC cells were counted and seeded onto a 6-well culture dish 5–10 × 10^5^ per well in 2 mL media. They were allowed to attach overnight and then treated as indicated. They were washed twice with PBS and lysed in RIPA buffer solution containing protease inhibitors. Protein lysates were loaded and electrophoresed on SDS-PAGE (6–16%) and were then transferred onto polyvinyl difluoride (PVDF) membranes (Millipore, Billerica, MA). Blocked with 5% non-fat milk, the PVDF membrane was incubated with various primary antibodies at 4 ℃ overnight, and subsequently incubated with corresponding secondary antibodies for 1 h. The protein–antibody complex was detected with a SuperSignal West Dura Extended Duration Substrate (Thermo Scientific, 34075; Waltham, MA) and visualized by the medical X-ray film (Carestream Health, Xiamen, China).

### c-Myc knockdown

Transfections were performed in 6-well dishes. siRNAs directed against human c-Myc and control RNAs were purchased from Viewsolid Biotech (China). Lipofectamine RNAiMAX (Invitrogen, 13778-150) was used to transfect, as per manufacturer’s instructions.

### Immunofluorescence (IF)

Cells grown on coverslips were treated with JQ1 or DMSO for 12 h. They were then fixed in 4% paraformaldehyde for 20 min, permeabilized with 0.3% Triton X-100 in PBS for 5 min, and incubated with the blocking solution (4% BSA in PBS) for 1 h. The cells were then incubated with the primary antibodies overnight at 4 °C, secondary antibodies and phalloidin (50 μg/ml) for 1 h, and DAPI for 10 min. The coverslips were mounted with slow fade Light Anti fade Kit (Invitrogen) and imaged with a Leica light microscope.

### RNA-seq

CNE2-EBV−/+ and TWO3-EBV−/+ cells were treated with 0.1% DMSO or 1 μM JQ1 for 3 h, and mRNA was extracted using an AllPure Cell Kit (Magen, Guangzhou, China) according to the manufacturer’s instructions and submitted to Beijing Novogene Bioinformatics Technology Company Limited for RNA-seq analysis. Analysis of RNA-seq data and gene set enrichment analysis (GSEA) were performed by Guangzhou Genedenovo Bio-Technology Company Limited. A detailed description of the GSEA methodology is provided at http://www.broadinstitute.org/gsea/doc/GSEAUserGuideFrame.html.

### Immunohistochemistry

Formalin-fixed, paraffin-embedded tissues of transplanted tumors were sectioned at 4 μm thickness. The sections were deparaffinized and stained with Ki-67 (CST; 12202S) or c-Myc (Santa Cruz; sc-40) followed by 3,3′-diaminobenzidine (DAB) staining. Primary antibodies were detected with a biotinylated secondary antibody. The sections were visualized under a light microscope.

### Quantitative reverse transcription PCR

qRT-PCR analysis was performed as previously described^41^. The primers used in this study are detailed in Table S2.

### Xenograft experiments

Female BALB/c nude mice (3–5 weeks old) were purchased from Beijing Vital River Laboratory Animal Technology Company Limited and were acclimated for 1 week. The mice were then injected subcutaneously with 1 × 10^6^ CNE2 cells or 2 × 10^7^ CNE2-EBV+ cells (16 mice each) in the right axillary cavity. When the tumors were palpable, the mice were randomized into two groups and treated twice a week with 50 mg/kg JQ1 or vehicle control in about 200 μL intraperitoneally for 2 weeks. Body weight and tumor volume (V) were measured every 2 days. Tumor volume was calculated from caliper measurements according to the equation *V* = (length × width^2^)/2. The mice were sacrificed when tumor volume reached 2000 mm^3^ or when the mice were in poor health. All animal work was performed according to the approved protocol of Sun Yat-sen University (No. L102042017040J).

### Statistical analysis and data

Data were analyzed with the GraphPad Prism 6 software. Two-tailed Student’s *t*-test was used to compare the statistical differences between groups. All P values of less than 0.05 were considered to be statistically significant. The authenticity of this article has been validated by uploading the key raw data onto the Research Data Deposit public platform (www.researchdata.org.cn), with the approval RDD number as RDDB2018000364.

## Electronic supplementary material

Supplemental material

## References

[1] Chua MLK, Wee JTS, Hui EP, Chan ATC (2016). Nasopharyngeal carcinoma. Lancet.

[2] Torre LA (2015). Global cancer statistics, 2012. CA Cancer J. Clin..

[3] Blanchard P (2015). Chemotherapy and radiotherapy in nasopharyngeal carcinoma: an update of the MAC-NPC meta-analysis. Lancet Oncol..

[4] Lee AW, Ma BB, Ng WT, Chan AT (2015). Management of nasopharyngeal carcinoma: current practice and future perspective. J. Clin. Oncol. Off. J. Am. Soc. Clin. Oncol..

[5] Zhang L (2016). Gemcitabine plus cisplatin versus fluorouracil plus cisplatin in recurrent or metastatic nasopharyngeal carcinoma: a multicentre, randomised, open-label, phase 3 trial. Lancet.

[6] Young LS, Yap LF, Murray PG (2016). Epstein–Barr virus: more than 50 years old and still providing surprises. Nat. Rev. Cancer.

[7] Holliday R (1989). DNA methylation and epigenetic mechanisms. Cell Biophys..

[8] Portela A, Esteller M (2010). Epigenetic modifications and human disease. Nat. Biotechnol..

[9] Arrowsmith CH, Bountra C, Fish PV, Lee K, Schapira M (2012). Epigenetic protein families: a new frontier for drug discovery. Nat. Rev. Drug Discov..

[10] Azad N, Zahnow CA, Rudin CM, Baylin SB (2013). The future of epigenetic therapy in solid tumours—lessons from the past. Nat. Rev. Clin. Oncol..

[11] Pfister SX, Ashworth A (2017). Marked for death: targeting epigenetic changes in cancer. Nat. Rev. Drug Discov..

[12] Gullo C, Low WK, Teoh G (2008). Association of Epstein–Barr virus with nasopharyngeal carcinoma and current status of development of cancer-derived cell lines. Ann. Acad. Med..

[13] Cheung ST (1999). Nasopharyngeal carcinoma cell line (C666-1) consistently harbouring Epstein–Barr virus. Int. J. Cancer.

[14] Filippakopoulos P, Knapp S (2014). Targeting bromodomains: epigenetic readers of lysine acetylation. Nat. Rev. Drug Discov..

[15] Zhu H (2016). BET bromodomain inhibition promotes anti-tumor immunity by suppressing PD-L1 expression. Cell Rep..

[16] Hsu C (2017). Safety and antitumor activity of pembrolizumab in patients with programmed death-ligand 1-positive nasopharyngeal carcinoma: results of the KEYNOTE-028 study. J. Clin. Oncol. Off. J. Am. Soc. Clin. Oncol..

[17] Belkina AC, Denis GV (2012). BET domain co-regulators in obesity, inflammation and cancer. Nat. Rev. Cancer.

[18] Filippakopoulos P (2010). Selective inhibition of BET bromodomains. Nature.

[19] Zuber J (2011). RNAi screen identifies Brd4 as a therapeutic target in acute myeloid leukaemia. Nature.

[20] Delmore JE (2011). BET bromodomain inhibition as a therapeutic strategy to target c-Myc. Cell.

[21] Mertz JA (2011). Targeting MYC dependence in cancer by inhibiting BET bromodomains. Proc. Natl. Acad. Sci. USA.

[22] Dawson MA (2011). Inhibition of BET recruitment to chromatin as an effective treatment for MLL-fusion leukaemia. Nature.

[23] Ott CJ (2012). BET bromodomain inhibition targets both c-Myc and IL7R in high-risk acute lymphoblastic leukemia. Blood.

[24] Chapuy B (2013). Discovery and characterization of super-enhancer-associated dependencies in diffuse large B cell lymphoma. Cancer Cell.

[25] Lockwood WW, Zejnullahu K, Bradner JE, Varmus H (2012). Sensitivity of human lung adenocarcinoma cell lines to targeted inhibition of BET epigenetic signaling proteins. Proc. Natl. Acad. Sci. USA.

[26] Shimamura T (2013). Efficacy of BET bromodomain inhibition in Kras-mutant non-small cell lung cancer. Clin. Cancer Res. Off. J. Am. Assoc. Cancer Res..

[27] Cheng Z (2013). Inhibition of BET bromodomain targets genetically diverse glioblastoma. Clin. Cancer Res. Off. J. Am. Assoc. Cancer Res..

[28] Segura MF (2013). BRD4 sustains melanoma proliferation and represents a new target for epigenetic therapy. Cancer Res..

[29] Asangani IA (2014). Therapeutic targeting of BET bromodomain proteins in castration-resistant prostate cancer. Nature.

[30] McCleland ML (2016). CCAT1 is an enhancer-templated RNA that predicts BET sensitivity in colorectal cancer. J. Clin. Investig..

[31] Shu S (2016). Response and resistance to BET bromodomain inhibitors in triple-negative breast cancer. Nature.

[32] Zhu X (2017). Bromodomain and extraterminal protein inhibitor JQ1 suppresses thyroid tumor growth in a mouse model. Clin. Cancer Res. Off. J. Am. Assoc. Cancer Res..

[33] Berenguer-Daize C (2016). OTX015 (MK-8628), a novel BET inhibitor, displays in vitro and in vivo antitumor effects alone and in combination with conventional therapies in glioblastoma models. Int. J. Cancer.

[34] Stine ZE, Walton ZE, Altman BJ, Hsieh AL, Dang CV (2015). MYC, metabolism, and cancer. Cancer Discov..

[35] Lin DC (2014). The genomic landscape of nasopharyngeal carcinoma. Nat. Genet..

[36] Zhou H (2015). Epstein–Barr virus oncoprotein super-enhancers control B cell growth. Cell host Microbe.

[37] Su X, Chakravarti D, Flores ER (2013). p63 steps into the limelight: crucial roles in the suppression of tumorigenesis and metastasis. Nat. Rev. Cancer.

[38] Abbas T, Dutta A (2009). p21 in cancer: intricate networks and multiple activities. Nat. Rev. Cancer.

[39] Hoos A (2016). Development of immuno-oncology drugs—from CTLA4 to PD1 to the next generations. Nat. Rev. Drug Discov..

[40] Casey SC (2016). MYC regulates the antitumor immune response through CD47 and PD-L1. Science.

[41] Li N (2016). A novel Smac mimetic APG-1387 demonstrates potent antitumor activity in nasopharyngeal carcinoma cells by inducing apoptosis. Cancer Lett..

