# Supplementary material for: BET bromodomain inhibitor JQ1 preferentially suppresses EBV-positive nasopharyngeal carcinoma cells partially through repressing c-Myc
Source: Cell Death Dis. 2018 Jul 9;9(7):761. doi: 10.1038/s41419-018-0789-1 (PMC6037792; doi:10.1038/s41419-018-0789-1)
Supplement: Supplementary file 1 — Supplemental material [file 41419_2018_789_MOESM1_ESM.docx]

**Supplementary Figure S1.**

The immortalized nasopharyngeal epithelial cell lines NP69 and N5-tert were treated with increasing concentrations of JQ1 and IC50 values were determined based on cell viability as measured by Cell-Titer GLO.


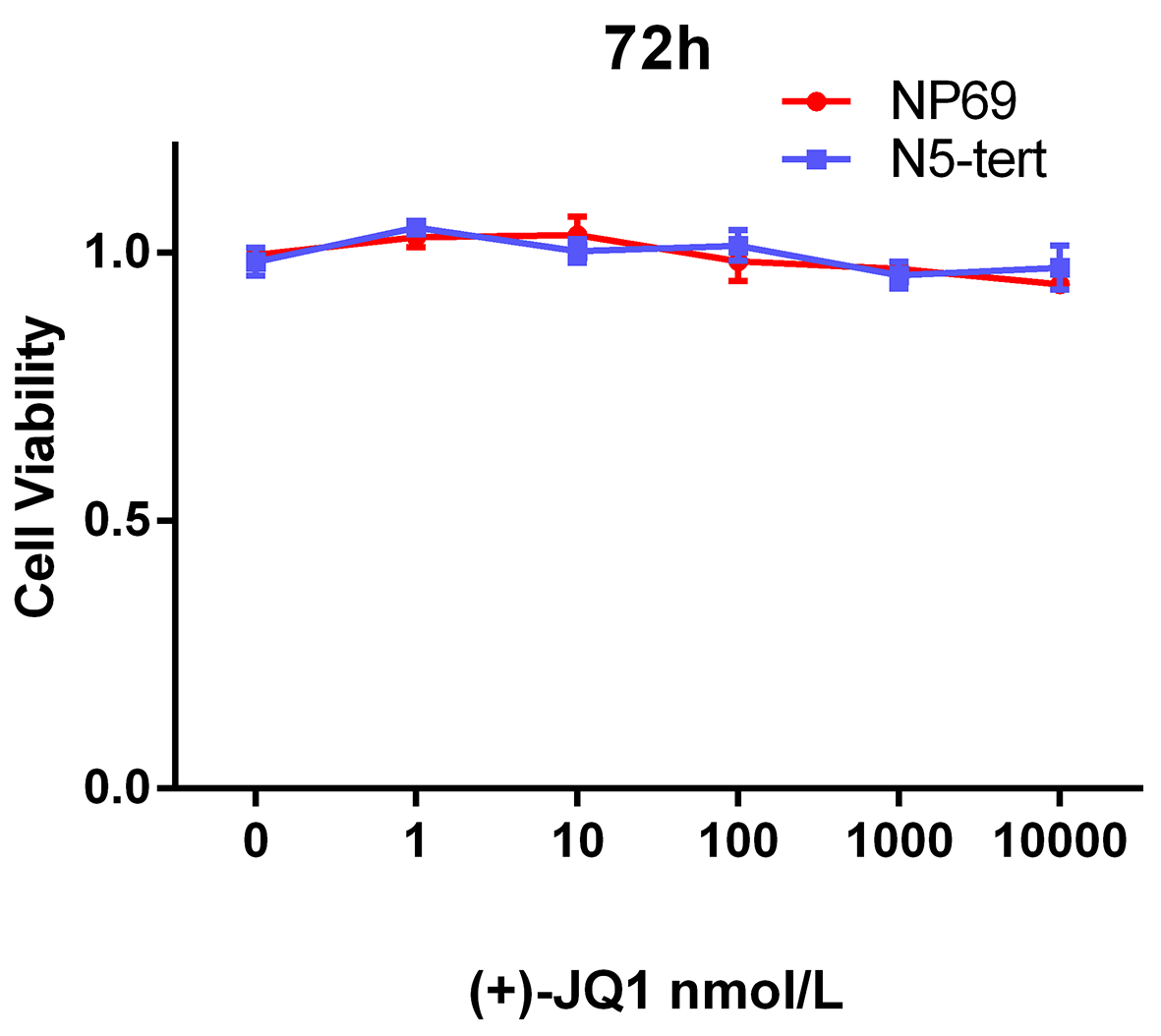


**Supplementary Figure S2.**

Annexin V and PI staining of CNE2-EBV-/+ cells treated with DMSO, JQ1 (500 nM), IR (4 Gy), or 500 nM JQ1 48 hours followed by IR of 4 Gy.


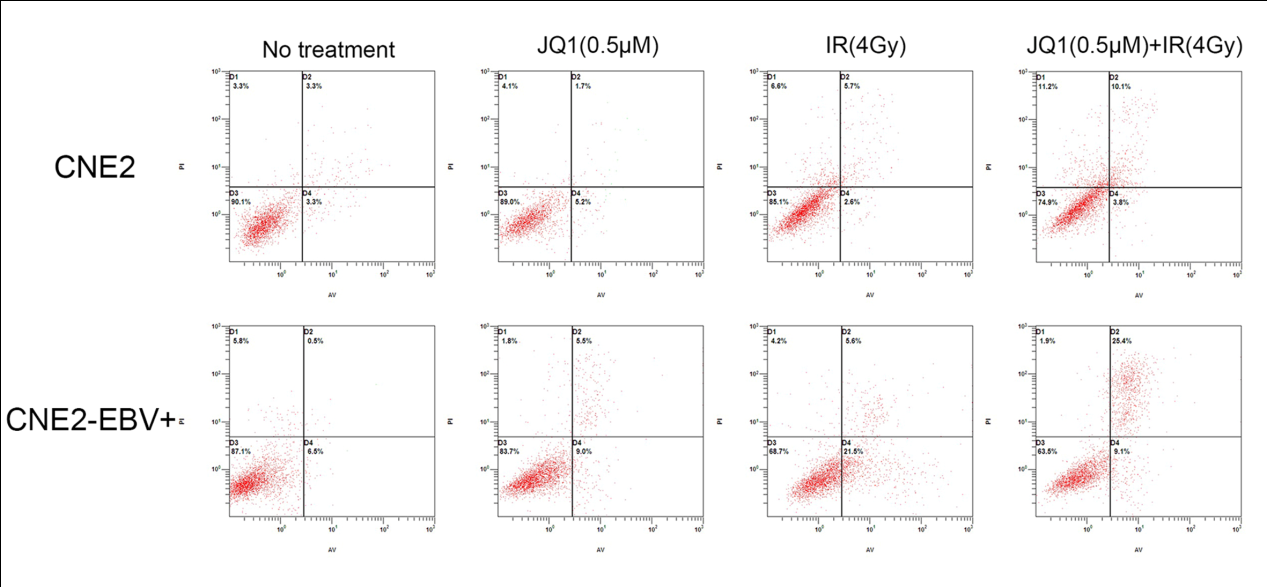


**Supplementary Figure S3.**

Heatmap representation of the top 30 differentially regulated genes between two EBV+ NPC cell lines (CNE2-EBV+ and TWO3-EBV+) and two EBV- NPC cell lines (CNE2 and TWO3) without JQ1 treatment (A) or with JQ1 treatment (B). Cells were treated with 1μM JQ1 for 3 hours.

**
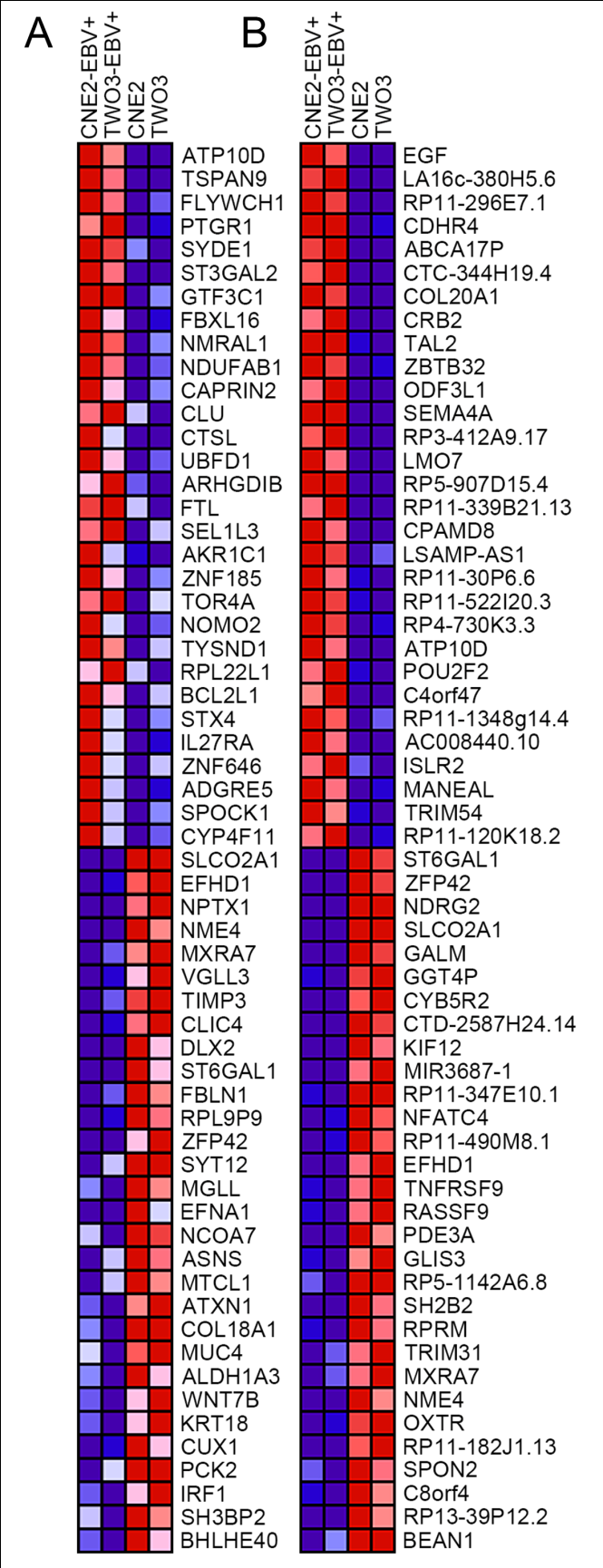
**

Table S1. The panel of 16 small-molecule inhibitors known to target epigenetic regulators used in this study.

| Number | Name | Target |
| --- | --- | --- |
| A4103 | LAQ824 | HDAC inhibitor |
| A8698 | SP-2509 | LSD1 inhibitor |
| B1583 | UNC1999 | EZH2 inhibitor |
| A4190 | GKS J4 | Inhibitor of H3K27 demethylase JMJD3 |
| S7498 | DDR1-IN-1 | Discoidin domain receptor 1 (DDR1) inhibitor |
| A1910 | JQ1 | BET bromodomain inhibitor |
| A4154 | Olaparib | PARP1/PARP2 inhibitor |
| B1577 | C646 | HAT p300-CBP inhibitor |
| B4891 | ML-324 | JMJD2 demethylase inhibitor |
| A4491 | SGC-CBP30 | Inhibitor of CREBBP/EP300 bromodomain |
| B5607 | IOX1 | histone demethylase JMJD inhibitor |
| S2003 | Maraviroc | CCR5 inhibitor |
| A3901 | UNC1215 | MBT (malignant brain tumor) antagonist |
| B4875 | PFI-2 | SETD7 methyltransferase inhibitor |
| B6091 | GSK-J1 | H3K27 histone demethylase inhibitor |
| B1582 | MM-102 | MLL1 inhibitor |

Table S2. The primers used in this study .

| Primer | Sequence |
| --- | --- |
| BRD2-forward | 5’-CTACGTAAGAAACCCCGGAAG-3’ |
| BRD2-reverse | 5’-GCTTTTTCTCCAAAGCCAGTT-3’ |
| BRD3-forward | 5’-CCTCAGGGAGATGCTATCCA-3’ |
| BRD3-reverse | 5’-ATGTCGTGGTAGTCGTGCAG-3’ |
| BRD4-forwrd | 5’-AGCAGCAACAGCAATGTGAG-3’ |
| BRD4-reverse | 5’-GCTTGCACTTGTCCTCTTCC-3’ |
